# Redirecting substrate regioselectivity using engineered ΔN_123_-GBD-CD2 branching sucrases for the production of pentasaccharide repeating units of *S. flexneri* 3a, 4a and 4b haptens

**DOI:** 10.1038/s41598-021-81719-1

**Published:** 2021-01-28

**Authors:** Mounir Benkoulouche, Akli Ben Imeddourene, Louis-Antoine Barel, Guillaume Le Heiget, Sandra Pizzut, Hanna Kulyk, Floriant Bellvert, Sophie Bozonnet, Laurence A. Mulard, Magali Remaud-Siméon, Claire Moulis, Isabelle André

**Affiliations:** 1 Toulouse Biotechnology Institute, TBI, Université de Toulouse, CNRS, INRAE, INSA, 135, avenue de Rangueil, 31077 Toulouse Cedex 04, France; 2 Unité de Chimie des Biomolécules, Institut Pasteur, UMR3523 CNRS, 28, rue du Dr Roux, 75724 Paris Cedex 15, France; 3 Université Paris Descartes, Sorbonne Paris Cité, Paris, France; 4 Université Paris 13, Bobigny, France; 5 MetaboHUB-MetaToul, National Infrastructure for Metabolomics and Fluxomics, Toulouse, France

**Keywords:** Carbohydrates, Enzymes, Glycobiology, Structural biology

## Abstract

The (chemo-)enzymatic synthesis of oligosaccharides has been hampered by the lack of appropriate enzymatic tools with requisite regio- and stereo-specificities. Engineering of carbohydrate-active enzymes, in particular targeting the enzyme active site, has notably led to catalysts with altered regioselectivity of the glycosylation reaction thereby enabling to extend the repertoire of enzymes for carbohydrate synthesis. Using a collection of 22 mutants of ΔN_123_-GBD-CD2 branching sucrase, an enzyme from the Glycoside Hydrolase family 70, containing between one and three mutations in the active site, and a lightly protected chemically synthesized tetrasaccharide as an acceptor substrate, we showed that altered glycosylation product specificities could be achieved compared to the parental enzyme. Six mutants were selected for further characterization as they produce higher amounts of two favored pentasaccharides compared to the parental enzyme and/or new products. The produced pentasaccharides were shown to be of high interest as they are precursors of representative haptens of *Shigella flexneri* serotypes 3a, 4a and 4b. Furthermore, their synthesis was shown to be controlled by the mutations introduced in the active site, driving the glucosylation toward one extremity or the other of the tetrasaccharide acceptor. To identify the molecular determinants involved in the change of ΔN_123_-GBD-CD2 regioselectivity, extensive molecular dynamics simulations were carried out in combination with in-depth analyses of amino acid residue networks. Our findings help to understand the inter-relationships between the enzyme structure, conformational flexibility and activity. They also provide new insight to further engineer this class of enzymes for the synthesis of carbohydrate components of bacterial haptens.

## Introduction

Carbohydrate-active enzymes catalyze a wide range of chemical reactions. They have emerged as a practical alternative to chemical catalysts, avoiding multiple steps of protection and deprotection often required in chemical synthesis to control the reactivity of the sugar hydroxyl groups and regio- and stereo-selectivity of the reaction. Some of them are rather versatile biocatalysts often displaying naturally a relaxed substrate specificity. This promiscuity can be further exacerbated by enzyme engineering to either broaden or narrow down the range of recognized substrates and/or control the reaction selectivity^1^. In particular, mutagenesis targeting the enzyme active site has been very efficient to alter substrate specificity and expand the structural diversity of the accessible glyco-products. In recent years, our group has been particularly active in this field, notably by engineering sucrose-active α-transglucosylases from Glycoside Hydrolase (GH) families 13 and 70 of the CAZy classification^2^, to produce a variety of carbohydrate derivatives and glycoconjugates^3–7^. In particular, the amylosucrase from *Neisseria polysaccharea*, a GH13 sucrose-active enzyme, was purposely tailored to act on non-natural substrates and produce glucosylated derivatives programmed to enter various chemo-enzymatic pathways combining in the most convenient manner chemical and enzymatic steps involved in the synthesis of antigenic oligosaccharides. In the continuity of these studies, we recently tested the ability of branching sucrases—native GH70 sucrose-active transglucosylases specialized in dextran branching—to regioselectively glucosylate a lightly protected tetrasaccharide (allyl α-l-rhamnopyranosyl-(1 → 2)-α-l-rhamnopyranosyl-(1 → 3)-(2-*O*-chloroacetyl-α-l-rhamnopyranosyl)-(1 → 3)-2-deoxy-2-trichloroacetamido-β-d-glucopyranoside, named herein **ABC′D′**), which was designed and chemically synthesized to serve as a common scaffold to precursors of *Shigella flexneri* serotype-specific haptens that could enter in the composition of a broad serotype coverage *Shigella* vaccine, while respecting criteria imposed by the enzyme plasticity. Indeed, the regioselective enzymatic α-d-glucosylation of **ABC′D′** led to several pentasaccharides, some of which featuring the glucosylation pattern of *Shigella flexneri* O-antigens^8^. While conversion was low, this promising achievement was the proof-of-concept that cutting-edge combination of a fine-tuned chemically synthesized **ABC′D′** tetrasaccharide scaffold and sucrose-active enzymes could be a way forward to a multivalent synthetic glycan-based vaccines. Obviously, further exploration of this original pathway aimed at a high yielding conversion of **ABC′D′** into selected monoglucosylated pentasaccharide building blocks of interest in the context of developing a *Shigella* vaccine was the next step. Altogether, six branching sucrases were assessed and found to mainly produce a monoglucosylated product called **P1**, harboring the specific α–(1 → 6) glucosylation pattern of *S. flexneri* serotype 4a/4b. Trace amounts of a second mono-glucosylated tetrasaccharide named **P2** that remained uncharacterized due to limited availability and a range of poly-glucosylated products were also formed. Among the tested enzymes, the most intriguing one was the so-called ΔN_123_-GBD-CD2 branching sucrase^9^, as it was the most efficient to glucosylate **ABC′D′**, although into a multitude of poly-glucosylated products in addition to **P1** and **P2**. Moreover, this enzyme is interesting as it is the only GH70 branching sucrase of known three-dimensional structure to date^10^. Detailed molecular dynamics through multi-level modelling methods revealed the high flexibility of several loops surrounding the catalytic pocket, which could play a beneficial role in the recognition of a broader range of acceptors^11^. To support this assumption, (semi)rational engineering of ΔN_123_-GBD-CD2 enabled the diversification of bulky flavonoid glucosides^6^ by use of a small mutant library targeting mutations four amino acid residues from acceptor subsites + 1 and + 2 of the catalytic pocket (Supplementary Fig. S1).

In light of these encouraging results, we undertook herein the screening of this focused library for the glucosylation of tetrasaccharide **ABC′D′** hoping that we could identify mutants with distinguished selectivity. Indeed, we hypothesized that the mutations introduced in the acceptor subsite could help to re-orient the catalytic productive binding of the tetrasaccharide, to diversify the glucosylation pattern or improve the yield of the two pentasaccharides originally synthesized by the parental enzyme. Upon screening, we identified mutants that produced two new compounds, pentasaccharide **P3** and hexasaccharide **H1**. Six mutants, containing single, double or triple substitutions were selected for further characterization as they produced higher amounts of **P1** and **P2** pentasaccharides than the parental ΔN_123_-GBD-CD2 branching sucrase and/or the new products **P3** and **H1**. The structure of pentasaccharide **P2** was characterized and shown to be characteristic of the *S. flexneri* 3a prevalent serotype^12^. The synthesis of both **P1** and **P2** showed that the introduced mutations drive glucosylation toward one extremity or the other of tetrasaccharide **ABC′D′** (**A** vs **D′** for **P2** and **P1**, respectively). To identify determinants possibly involved in the control of the regioselectivity, we used Molecular Dynamics (MD) simulations combined with in-depth analyses of amino acid residue networks.

This study highlights the potential of GH70 branching sucrase engineering for glucodiversification of a *S. flexneri* tetrasaccharide backbone as well as to control the reaction regioselectivity. These promising results pave the way for a more exhaustive reshaping of branching sucrase active site to further extend the panel of accessible glucosylation patterns and ultimately, provide synthetic tools for the development of a broad coverage synthetic glycan-based vaccine against *S. flexneri*.

## Results and discussion

### Screening of the mutant library

**Figure 1:**
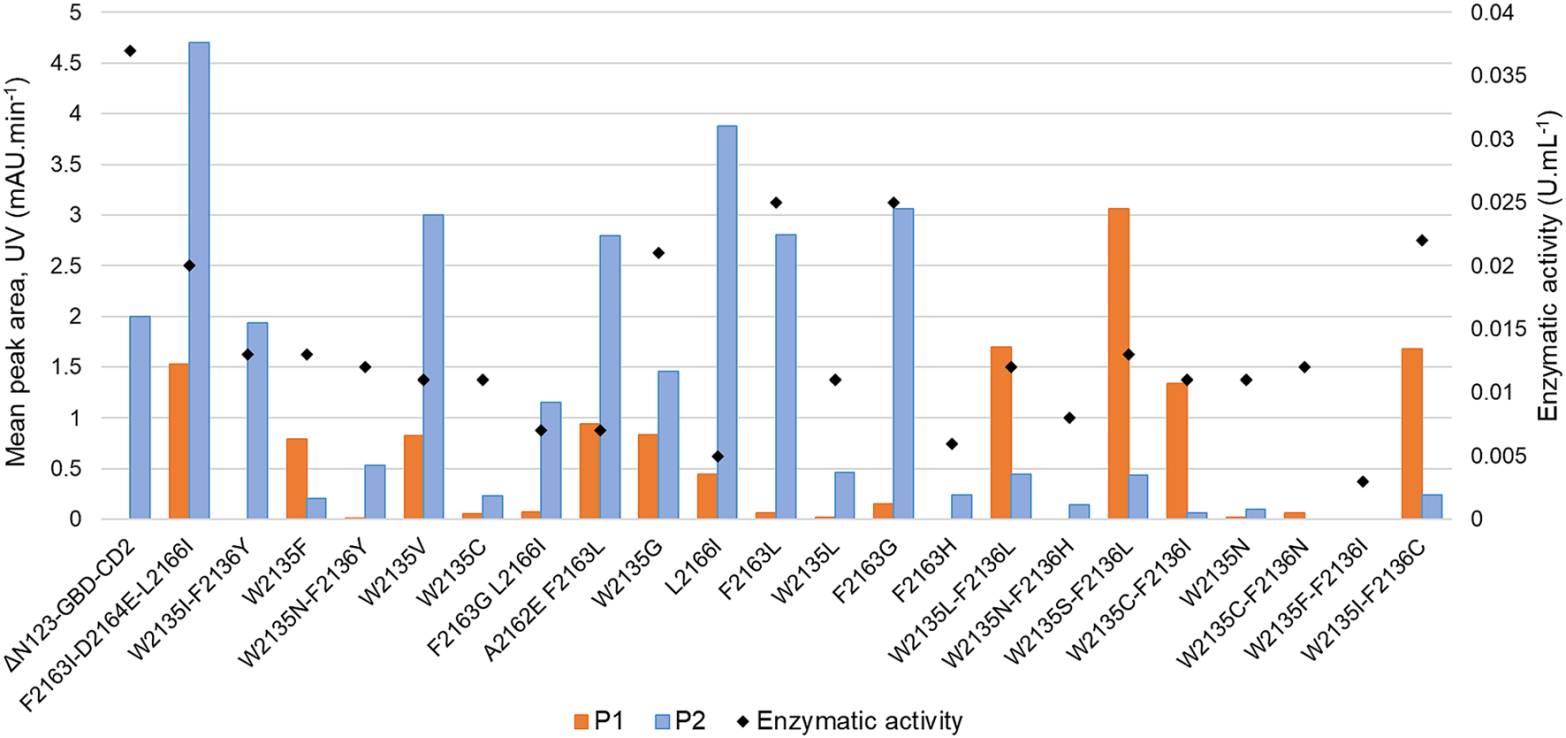
Amounts of products **P1** (orange) and **P2** (blue) formed with ΔN_123_-GBD-CD2 branching sucrase and its mutants in screening conditions. Reactions were carried out in 10 µL volume with 1 M sucrose and 50 mM **ABC′D′** for 16 h at pH 4.7 and 30 °C, using between 0.003 and 0.037 U mL^−1^ enzymatic activity (black diamonds). Quantities were estimated from mean peak areas obtained by HPLC–UV analysis at 220 nm.

**Figure 2:**
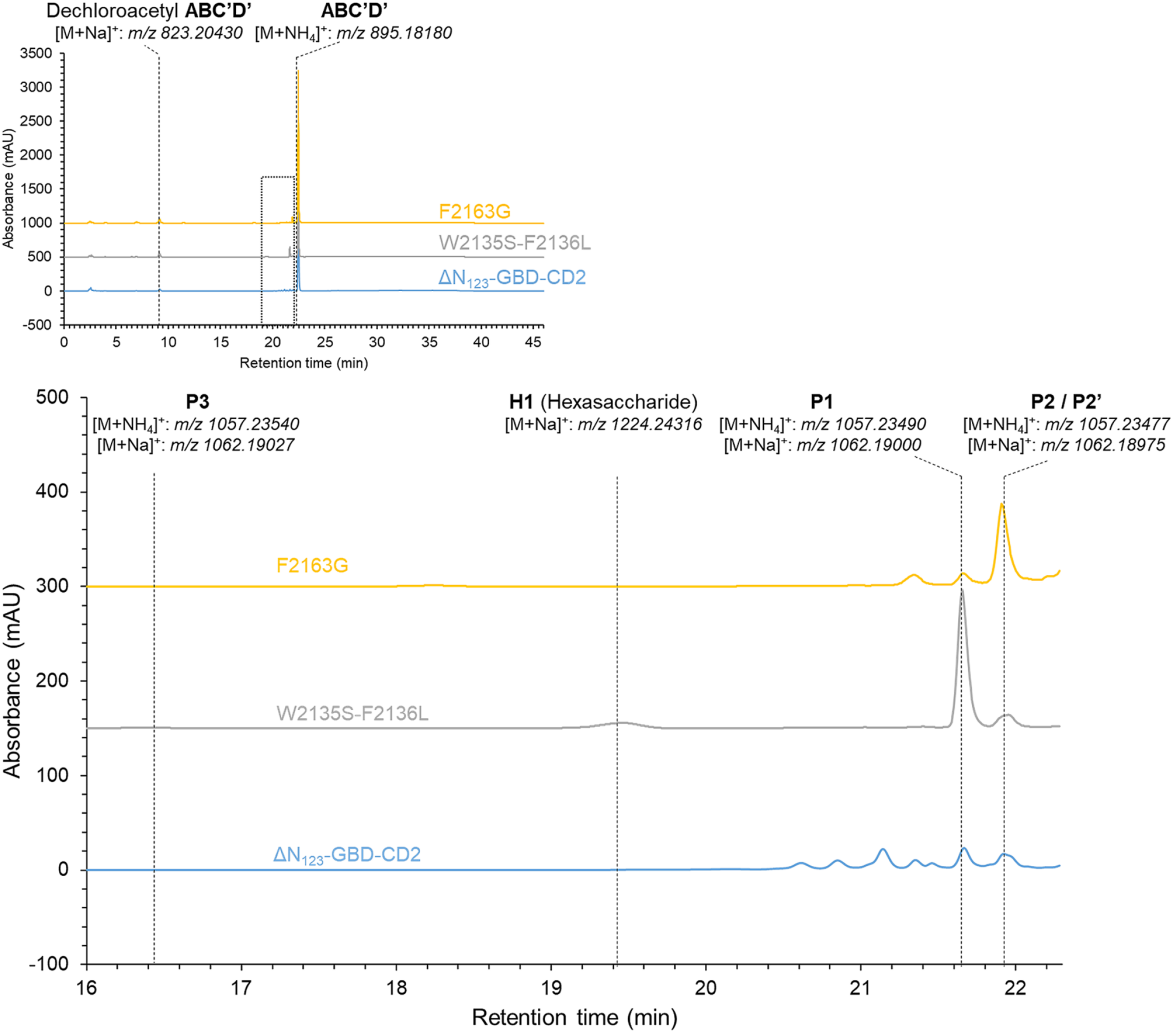
HPLC-UV_220nm_ and HRMS analysis of glucosylation products obtained with ΔN_123_-GBD-CD2 branching sucrase and its mutants, W2135S-F2136L and F2163G after 16 h of reaction in the presence of sucrose (1 M) and tetrasaccharide **ABC′D′** (50 mM) at pH 5.1 using 1 U mL^−1^ enzyme and 30 °C. A zoom of the product region located between 16 and 22.3 min is shown. **P1** (*t*_*r*_ = 21.65 min), **P2/P2′** (*t*_*r*_ = 21.9 min), **P3** (*t*_*r*_ = 16.4 min) and **H1** (*t*_*r*_ = 19.45 min). A shift in molecular mass by 162 Da, characteristic of monoglucosylation, was observed for **P1**, **P2/P2′** and **P3** and a shift of 362 Da (di-glucosylation) was observed for **H1** (*m/z* detected by HRMS indicated below each product and corresponding to NH4^+^ or Na^+^ adducts in positive mode).

The library of 22 variants, generated from ΔN_123_-GBD-CD2 branching sucrase^6^ and comprising enzymes previously shown to exhibit a good ability to recognize bulky acceptor substrates, was screened for the glucosylation of tetrasaccharide **ABC′D′**. To limit the quantity of tetrasaccharide used, the reaction volume was scaled down to 10 µL and the reaction pH was fixed at 4.7 to avoid degradation of the chloroacetyl protecting group on **C′** unit^8^. For all enzymes, the activity values on sucrose alone were lower at pH 4.7 than at pH 5.75 (the optimal pH of the parental enzyme^13,14^) but remained sufficiently high to allow screening (data not shown)**.** With the exception of mutant W2135F-F2136I (the least active mutant on sucrose alone at pH 4.7), all enzymes were able to glucosylate tetrasaccharide **ABC′D′** (present at 50 mM) and 1 M of sucrose. LC/MS analysis^8^ revealed that the two previously reported monoglucosylated tetrasaccharides **P1** and **P2** obtained with the parental enzyme (retention times 21.65 min and 21.9 min, respectively, and mass values *m/z*: 1057.23596 and 1057.23564, respectively) were produced in varying amounts depending on the enzyme (Figs. 1, 2). Of note, in these screening conditions, ΔN_123_-GBD-CD2 branching sucrase produced only **P2** while we showed that it synthesized **P1**, **P2** and several poly-glucosylated products using reaction higher pH and volume^8^.

Comparison of the product profiles allowed to distinguish three categories. The first one gathers 6 mutants (W2135F, W2135L-F2136L, W2135S-F2136L, W2135C-F2136I, W2135C-F2136N and W2135I-F2136C), that synthesize higher amounts of **P1** than **P2**. Conversely, the second group comprises 6 mutants (F2163L-D2164E-L2166I, W2135V, A2162E-F2163L, L2166I, F2163G and F2163L) that mainly produce **P2** and in higher amounts than ΔN_123_-GBD-CD2. In the third group are the 9 remaining mutants showing a product profile similar to that of the parental enzyme (mutant W2135I-F2136Y) or being poorly active on tetrasaccharide **ABC′D′**.

The residues W2135, F2136 and L2166 (in the first shell of the acceptor subsites + 1/ + 2) and F2163 (in the second shell) all belong to the domain B of ΔN_123_-GBD-CD2^9^ (Supplementary Fig. S1). In the seminal work that led to the construction of the mutant library, they were targeted with the aim of gaining space to better accommodate bulky flavonoid molecules in the acceptor subsites^6^. We hypothesized that the same mutations could also help to accommodate the large tetrasaccharide **ABC′D′,** which harbors bulky protecting groups at positions 1_D′_, 2_D′_ and 2_C′_, in a catalytically productive manner. It turned out that substituting the cumbersome residues W2135 and F2136 by smaller residues such as leucine or serine favored formation of **P1**. Conversely, the substitution of F2163 by aliphatic and more flexible residues (glycine, isoleucine, leucine) favored formation of **P2.** In contrast, the introduction of other bulky residues such as histidine (mutants W2135N-F2136H or F2163H) led to formation of glucosylated products in lower amounts. Altogether, these results reveal the possibility to modulate the production of **P1** and **P2** depending on the mutations introduced in the active site.

To confirm the product profiles, we selected the best producers from category 1 (W2135S-F2136L, W2135I-F2136C and W2135L-F2136L) and category 2 (F2163G, L2166I and F2163L-D2164E-L2166I) and carried out the glucosylation experiment in 50 µL reaction volume, at pH 5.1, which is a better pH compromise between enzyme activity and tetrasaccharide stability, with 1 U mL^−1^ enzyme, 50 mM of tetrasaccharide **ABC′D′** and 1 M of sucrose. The products were analyzed using LC/HRMS The best representatives of category 1 and 2 are shown in Fig. 2.

**Figure 3:**
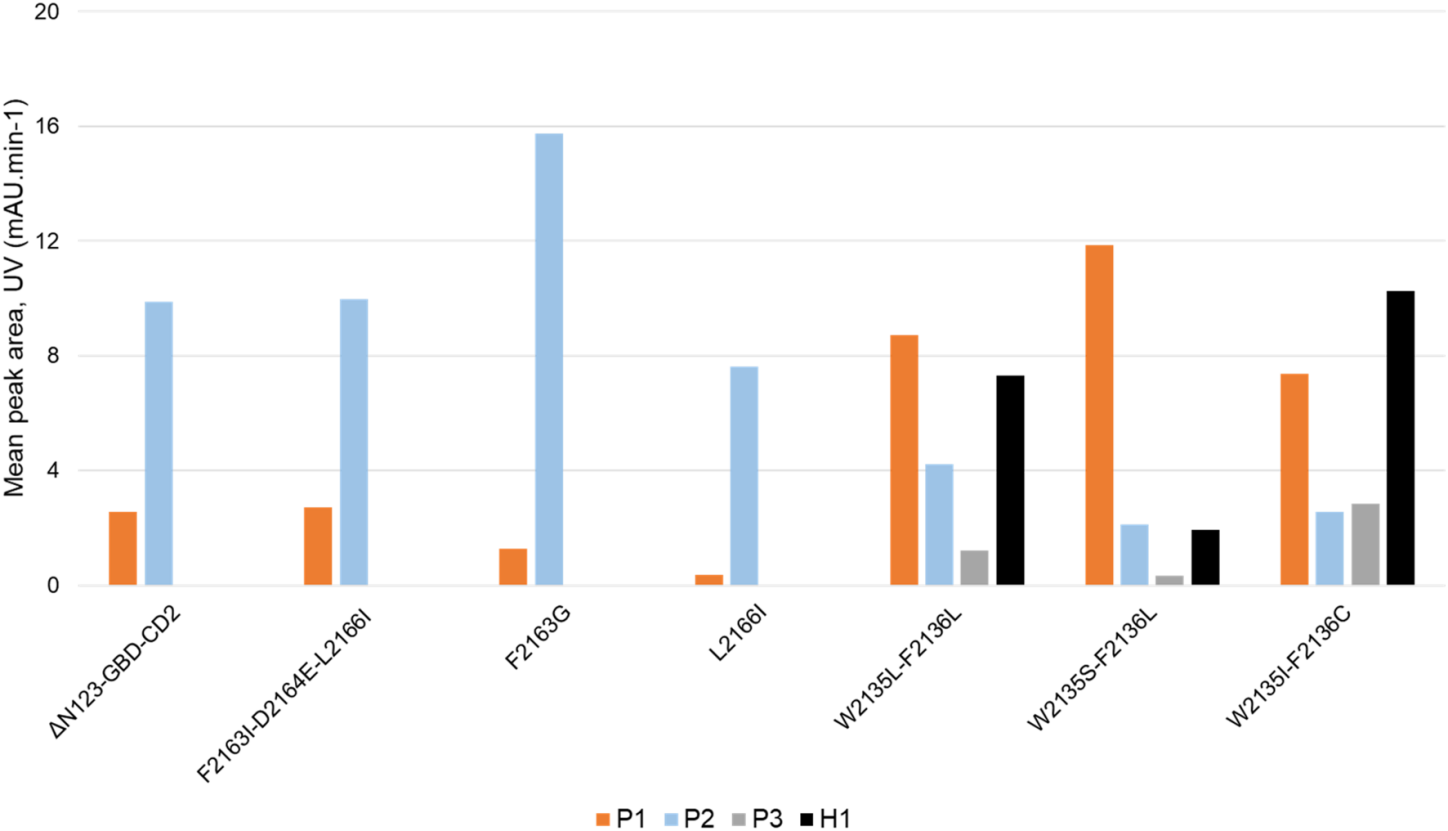
Distribution of products formed by ΔN_123_-GBD-CD2 branching sucrase and the six selected mutants (F2136I-D2164E-L2166I, F2163G, L2166I, W2135L-F2136L, W2135S-F2136L and W2135I-F2136C). The bar plot gives the amount of pentasaccharides [**P1** (orange), **P2** (cyan), **P3** (gray)] and the hexasaccharide (**H1** black) as detected by HPLC–UV (wavelength at 220 nm). Reactions were performed in the presence of 1 M sucrose and 50 mM tetrasaccharide **ABC′D′**, for 16 h using 1 U mL^−1^ enzymes at pH 5.1 and 30 °C.

In these conditions, the product profile of ΔN_123_-GBD-CD2 branching sucrase revealed the presence of small amounts of **P1** ([M + Na]^+^: *m/z* 1062.19000) and polyglucosylated products as previously reported in similar reaction conditions^8^. We confirmed that the three enzymes mutated at positions 2135–2136 catalyzed mainly **P1** formation, a smaller amount of **P2** ([M + Na]^+^: *m/z* 1062.18975) and traces of a third monoglucosylated product **P3** ([M + Na]^+^: *m/z* 1062.19027)**,** not observed in reactions performed at pH 4.7 in 10 µL volume but synthesized in too small quantities for NMR structure elucidation. A hexasaccharide, named **H1** ([M + Na]^+^: *m/z* 1224.24316), also never identified before was detected (*t*_*r*_ = 19.45 min, Fig. 2). It remains unclear whether this double glucosylation occurred at the same or distinct positions. Interestingly, the polyglucosylated products produced by the parental enzyme with up to 6 glucosyl units transferred were not produced by the mutants (Fig. 2). Regarding the three other mutants targeting positions 2163 and 2166, we confirmed their inclination to produce preferentially **P2**, although only mutant F2163G was finally able to outperform parental ΔN_123_-GBD-CD2 branching sucrase (Fig. 3).

### Structural characterization of the novel pentasaccharides

**Figure 4:**
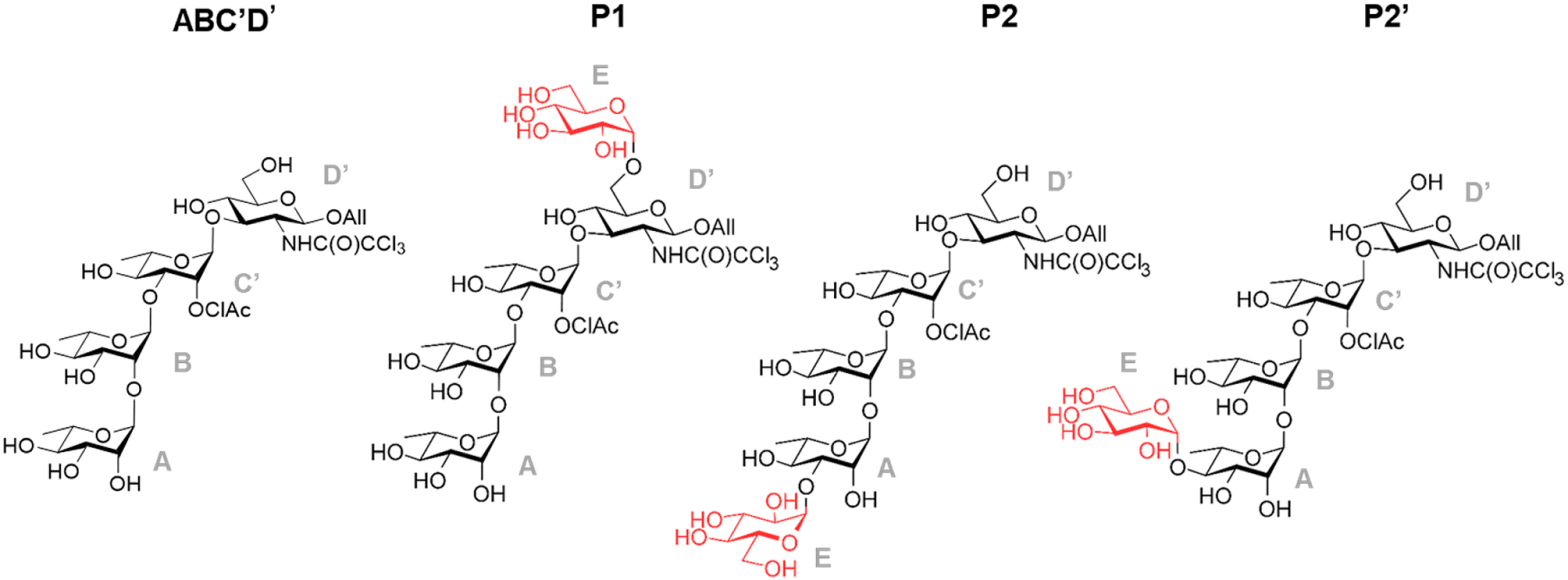
Structure of the tetrasaccharide acceptor (**ABC′D′**) and pentasaccharide products **P1** (determined in Ref.^8^, **P2** and **P2′** (**E(1 → 3)ABC′D′** and **E(1 → 4)ABC′D′**, respectively) characterized by NMR spectroscopy. In red is highlighted the glucosyl moiety (**E**). *All *allyl, *ClAc *chloroacetyl. Drawing of chemical structures done using CHEMDRAW (PerkinElmer).

F2163G mutant was purified and used to produce **P2** in suitable amounts for structural characterization. Reaction products were isolated and analyzed by HPLC–UV-HRMS, which revealed that **P2** was in fact a mixture of two co-eluted products that we called respectively **P2** and **P2′.** About 5 mg of **P2′** in pure form could be obtained upon second purification (4.8% global yield (mol/mol) corresponding to the enzymatic conversion and the two subsequent steps of purification) that allowed its structure elucidation by NMR analysis in combination with previous resonance assignments of **ABC’D’** NMR spectra^8^. Data showed that glucosylation of **ABC′D′** occurred at OH-4_A_, leading to allyl α-d-glucopyranosyl-(1 → 4)-α-l-rhamnopyranosyl-(1 → 2)-α-l-rhamnopyranosyl-(1 → 3)-2-*O*-chloroacetyl-α-l-rhamnopyranosyl-(1 → 3)-2-deoxy-2-trichloroacetamido-β-d-glucopyranoside, named **E(1 → 4)ABC′D′**, or **P2′** in the HPLC profiles (Fig. 2). Then, NMR analysis of the mixture of **P2** and **P2′** also comprising a dechloroacetylated degradation product, allowed to deduce that **P2** was glucosylated at OH-3_A,_ corresponding to allyl α-d-glucopyranosyl-(1 → 3)-α-l-rhamnopyranosyl-(1 → 2)-α-l-rhamnopyranosyl-(1 → 3)-2-*O*-chloroacetyl-α-l-rhamnopyranosyl-(1 → 3)-2-deoxy-2-trichloroacetamido-β-d-glucopyranoside, also named **E(1 → 3)ABC′D′**, or **P2** in the HPLC profiles (Fig. 4).

In details, analysis of the HSQC spectra showed shifted resonances of the glucosylated positions and adjacent atoms (Supplementary Fig. S2); the glucosylated 4_A_ carbon was shifted toward higher frequency from 72.2 to 81.2 ppm, while the adjacent 3_A_ and 5_A_ carbons were shifted to lower frequency, from 70.1 ppm and 69.2 ppm to 68.3 ppm and 68.4 ppm, respectively. The cross-correlation peaks between shifted resonances were assigned using Heteronuclear Multiple-Bond Correlation spectroscopy (HMBC) experiments (Supplementary Fig. S2C), and the Double Quantum Filtered COrrelation SpectroscopY (DQF COSY)^15^ experiments (Supplementary Fig. S3).

The resonance assignments of **P2** were extracted from the NMR spectra of the mixture of products (referenced below as a mixture including pentasaccharides **P2** and **P2′**, as well as at least one dechloroacetylated pentasaccharide) and was performed firstly by the subtraction of signals known to belong to the spectra of the **P2′** isomer (Supplementary Fig. S4A). The **E** units belonging to **P2** resonance was found by subtraction of **P2′** and dechloroacetylated tetrasaccharide signals. All connectivities of the latter **E** units were found by DQF COSY spectrum (Supplementary Fig. S5). The assignment of the glucosylated position 3_A_ was derived from the HSQC spectrum. Only the resonance of 3_A_ from the non-superimposed tetrasaccharide peaks, was shifted, the 4_A_ and 4_B_ were superimposed in the tetrasaccharide spectrum. This observation suggested two assumptions; the glucosylation occurred on 3_A_ or on 2_A_ of the tetrasaccharide. It is known that glucosylation leads to slightly lower frequency shift of the adjacent resonance of the glucosylated position. However, no peak was observed in the anomeric region (Supplementary Fig. S4B), which confirmed that glucosylation occurs at OH- 3_A_. The HMBC spectrum showed the cross-correlation peak of the **E** units and the assigned 3_A_ resonance.

### Structural insight on the impact of the mutations on the regioselective glucosylation of tetrasaccharide **ABC′D′**

**Figure 5:**
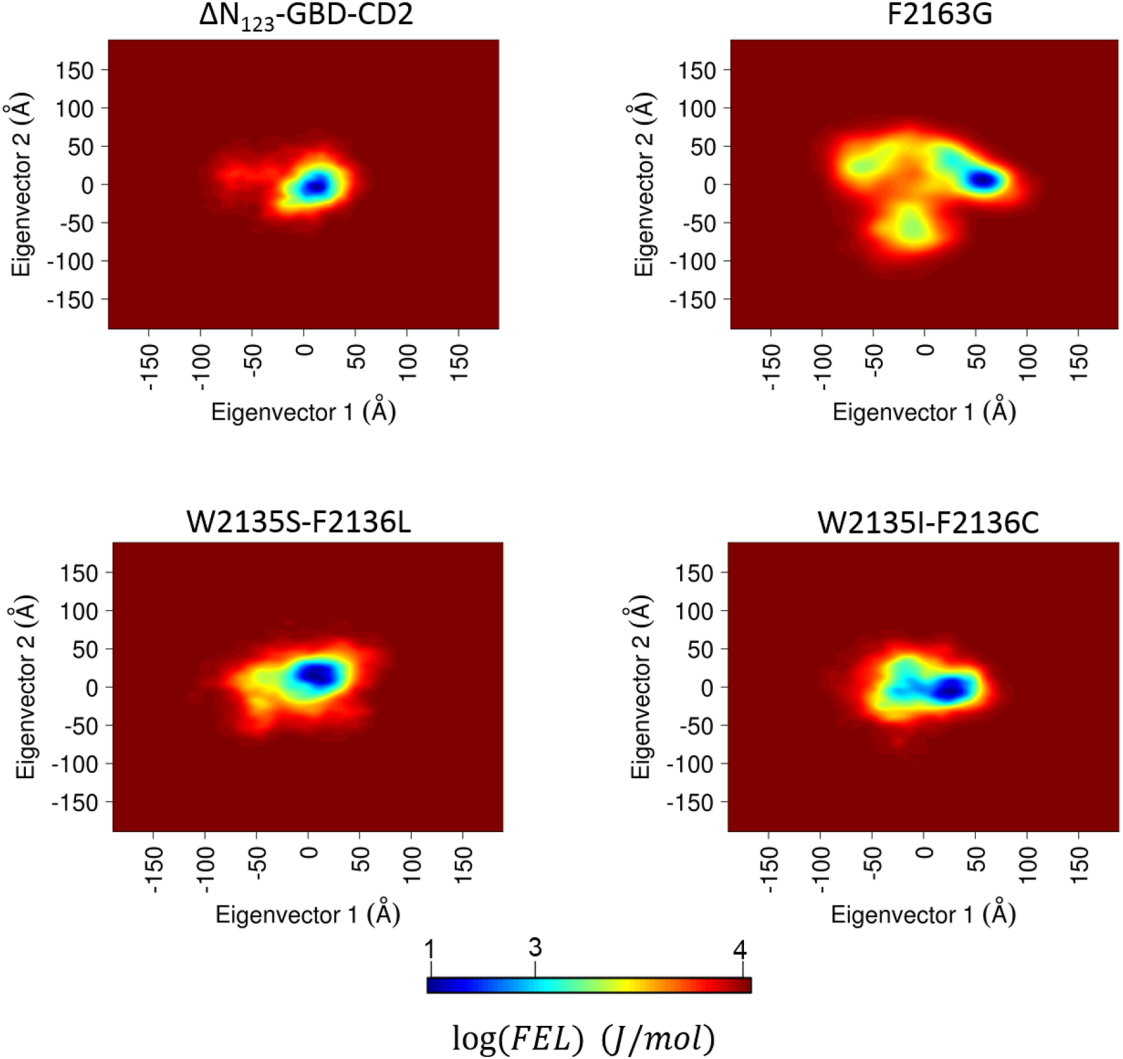
Free energy landscapes (FELs) of ΔN_123_-GBD-CD2, F2163G, W2135I-F2136C and W2135I-F2136C were determined using as reaction coordinates the projection of the first and second principal components from one microsecond MD simulation of the free ligand form for each system. The bottom legend shows the color scale of the logarithm of FEL in J mol^−1^.

Results described above revealed that glucosylation can occur either on the **D′** (**P1**) or **A** (**P2**) residue of the tetrasaccharide depending on the mutations introduced in the active site of ΔN_123_-GBD-CD2 branching sucrase. This suggests that **ABC′D′** can bind in at least two distinct modes in the active site and still be glucosylated by the enzyme. We used computational methods to further investigate the versatile binding modes of this non-natural chemically designed tetrasaccharide acceptor at an atomic level and to understand the influence of the mutations on activity toward sucrose donor and on the selective glucosylation of the acceptor substrate (Fig. 4). In particular, MD simulations^16–25^ (of various lengths: 1 µs, 100 ns and 2 ns) were carried out in free form or in complex^26^ with sucrose or products **P1** or **P2** that were constructed and parametrized in silico^27–30^, for parental ΔN_123_-GBD-CD2 branching sucrase, and the modelled^31^ mutants F2163G, W2135S-F2136L, and W2135I-F2136C. The free energy landscapes (FELs) from the two first eigenvectors of each 1 μs MD simulation reflect the structure and dynamic perturbation due to the mutations on the global motion of the enzymes (Fig. 5)^11^. Interestingly, the mutants W2135S-F2136L, and W2135I-F2136C showed a high resemblance in their FEL; the lowest energy basins shifted to the positive value for the first eigenvector. Furthermore the negative regions of this eigenvector were more populated than the parental enzyme, while the second component does not seem affected by the mutations. The F2163G mutant exhibited the broadest FEL indicating the high flexibility of this mutant. The principal component analyses, performed on the whole enzymes provided the trend of the global dynamics modes. However, to understand the impact of the mutations on the product profiles, further analyses focused on the active sites needed to be carried out. Thus, MD simulations were analyzed using Fruchterman–Reingold algorithm^32^ and the spin-glass algorithm^33^ to identify the amino acid network in the active site and define corresponding amino acid clusters (also called communities). Network analysis is often used in biology to understand cell functional organization via protein network or to clusterize metagenomics sequences. Network analysis derived from MD simulations (correlation matrix) were previously used to identify with success the signaling pathways in bacterial glutamyl-tRNA synthetase (GluRS):tRNA^Glu^ and an archaeal leucyl-tRNA synthetase LeuRS):tRNA^Leu^ complexes^34^. In the current study, we used graph theory to map the distance matrices of the active site residues^35^ and examine the effect of the mutation on the catalytic pocket topology, and possibly relate it to the product profile. To our knowledge, this is the first time such approaches were used in this context, providing essential information on the catalytic behavior of this family of enzymes.

**Figure 6:**
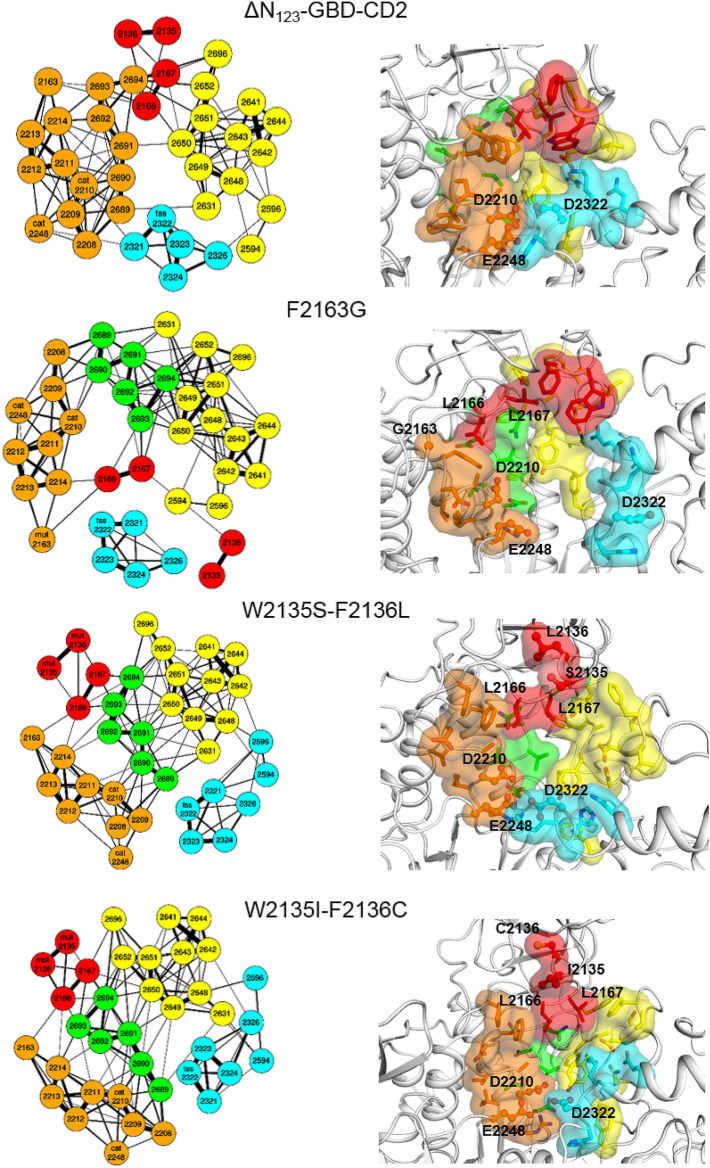
Comparison of network communities (left) and structural representation (right) derived from MD analysis. Left: Active site residues network presented by spring layout using Fruchterman-Reingold algorithm from distance matrixes from 1 µs MD simulations of the parental ΔN_123_-GBD-CD2, the F2163G mutant, and the double mutants W2135S-F2136L and W2135I-F2136C. Amino acid communities highlighted with orange, red, green, yellow and cyan colors were found by spin-glass algorithm. The catalytic, the transition state stabilizer, and the mutated residues are labelled respectively by “cat”, “tss” and “mut” for each corresponding graph. Right: View of active site residue clusters from the spin-glass algorithm for the parental ΔN_123_-GBD-CD2 and the mutants F2136G, W2135S-F2136L, and W2135I-F2136C. The 5 communities are presented: I (orange), II (green), III (red), IV (yellow) and V (cyan). Three dimensional structures are taken from the low energy regions of the free energy landscapes of each system (Fig. 5). Graphs in figures were drawn using the R software^39^. Molecular graphics were prepared using PYMOL 1.7 (PyMOL Molecular Graphics System, Schrödinger, LLC).

Except for the parental enzyme, the clustering algorithm found 5 communities [I (orange), II (green), III (red), IV (yellow) and V (cyan)] (Fig. 6); for all mutants the residues 2689–2694 form the cluster II, while the clusters I (orange) and II (green) were gathered in one single large cluster in the case of the parental enzyme. The F2163G mutation weakened the interaction inside the community III compared to the parental enzyme and other mutants. Furthermore, the cluster III in the case of the F2163G mutation had a different localization, nearby the cluster V. Remarkably, this proximity between these two communities situated on both sides of the active site led to the clear separation of clusters I and IV (Fig. 6). Interestingly, mutants W2135S-F2136L and W2135I-F2136C, which display the same product profile, showed a high similarity in their networks and community interactions: (i) all the vertices of the cluster II are linked to the two clusters I and IV; (ii) all nodes belonging to the cluster III are connected to each other with the exception of S2135 and L2167 residues in the case of mutant W2135S-F2136L; (iii) the two leucines 2166 and 2167 from cluster III bridged the clusters I, II and IV except the L2167 residue in the case of mutant W2135S-F2136L; (iv) the community V is connected to cluster IV and not to cluster I.

In the present study, the population shifts observed in Fig. 6 could help to explain the variability of product profiles between parental enzyme and its mutants. Furthermore, our graph analysis illustrates the thermodynamical displacement toward one conformation or another among the different mutants. Preexistence of such conformation changes is also underlined by the correlation found between the network derived from MD simulations and the product profiles of mutants W2135S-F2136L and W2135I-F2136C obtained from distinct acceptors (**ABC′D′** tetrasaccharide studied herein, Fig. 1, and flavonoids^6^).

**Figure 7:**
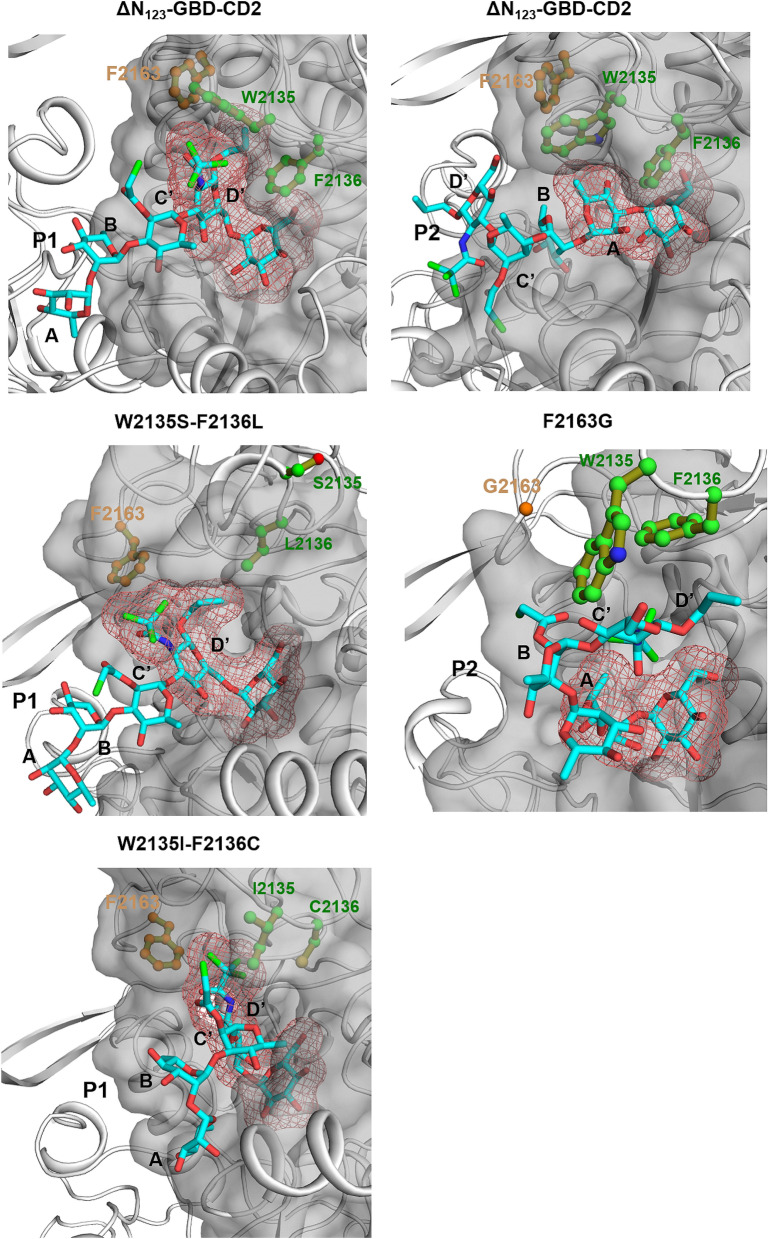
View of the active sites of mutants W2135S-F2136L and W2135I-F2136C in complex with their preferred product **P1 **(left) and mutant F2163G in complex with favored product **P2 **(right). Parental ΔN_123_-GBD-CD2 is shown in presence of **P1** and **P2** for reference purpose. The 3D coordinates were taken from the last frames of the simulated annealing pursued by 2 ns MD simulation of each complex. Molecular graphics were prepared using PYMOL 1.7 (PyMOL Molecular Graphics System, Schrödinger, LLC).

Active site networks are represented on 3D structures of the free ligand forms of the enzymes (Fig. 6) and mutations are highlighted in the modelled complexes with pentasaccharides (Fig. 7). Mutation of the bulky residues W2135 and F2136 clearly results in a gain of space that facilitates the accommodation of the bulky protecting groups of residue **D′** and allowed the acceptor to be oriented with the OH-6_D’_ in a conformation more catalytically favorable to produce **P1** (Fig. 7). The higher amount of **P1** obtained for variant W2135S-F2136L in comparison to variant W2135I-F2136C (Fig. 3) could result from the re-orientation of S2135 residue toward the solvent (Fig. 7), further widening the + 1 subsite. This conformation is reflected by the absence of edge between the S2135 and L2167 mentioned above (Fig. 6).

The flexibility of the loops surrounding the active site in the mutant F2163G was increased due to the introduction of the glycine residue that could destabilize the interactions mediated by this F2163 residue and thus leading to variations of the RMSD and high B-factor values observed for F2163G mutant compared to parental enzyme (Supplementary Fig. S6A,B). The population shift in this case led to more proximity between the clusters III and V. In this topological organization, the displacement of the loop containing residues W2135 and F2136 toward community V disfavors the accommodation of the tetrasaccharide with **C′** and **D′** moieties in a productive conformation for **P1** production. Indeed, the simulation illustrates the closeness of the loop containing the residues W2135 and F2136 to L2166 and L2167 from cluster V, promoting a bent conformation of the pentasaccharide, with the **D′** well stacked to the W2135 and F2136 residues through strong C–H…π interactions. These favorable interactions could compensate for the entropy loss due to this conformation bending in comparison to the parental enzyme for which the **C′** and **D′** moieties are found rather exposed to the solvent. Altogether, this could help to improve glucosylation of the **A** moiety in mutant F2163G.

### Effect of mutations on the sucrose kinetics of mutants

To investigate further the effect of mutations on sucrose utilization, we determined kinetic parameters on sucrose of the parental ΔN_123_-GBD-CD2 branching sucrase and its mutants F2163G and W2135S-F2136L. Mutant W2135I-F2136C was discarded from this evaluation as it displayed the same profile as W2135S-F2136L. When sucrose is used as sole substrate, the parental ΔN_123_-GBD-CD2 enzyme and its mutants displayed Michaelis–Menten kinetics for sucrose hydrolysis^36^ (Supplementary Fig. S8). The K_M,_ V_max_ and k_cat_ values obtained at pH 5.1 (30.47 ± 7.22 mM, 77.50 ± 6.09 μmol min^−1^ mg^−1^) are different from those that we previously reported for ΔN_123_-GBD-CD2^10^ and that were obtained at pH 5.4 (7.5 mM, 36.3 μmol min^−1^ mg^−1^). This may be an effect of pH change but the comparison is difficult as in our previous study the enzyme purification was performed differently by renaturation of unfolded proteins extracted from inclusion bodies. However, when comparing the kinetic parameters of ΔN_123_-GBD-CD2 (given herein) with the mutants F2163G and W2135S-F2136L, we note a ~ 3.5-fold decrease of K_M_ and a 4.5-fold increase of V_max_ indicating that the mutations have affected both parameters, but without profound improvements or deleterious effects (Table 1).

**Table 1:** Determination of kinetic parameters (K_M_, V_max_ and k_cat_) for sucrose (suc) hydrolysis of ΔN_123_-GBD-CD2 parental enzyme and mutants F2163G and W2135S-F2136L.

	ΔN_123_-GBD-CD2	W2135S-F2136L	F2163G
K_M,suc_ (mM)	30.5 ± 7.2	8.8 ± 1.0	7.0 ± 1.2
V_max, suc_ (µmol min^−1^ mg^−1^)	77.5 ± 6.1	17.7 ± 0.4	16.8 ± 0.5
k_cat, suc_ (s^−1^)	161.7 ± 12.7	36.9 ± 0.9	35.1 ± 0.9

Values were obtained by the least square fit of the Michaelis–Menten equation (Supplementary Table S1) and using increasing concentrations of sucrose (ranging from 10 to 300 mM), at pH 5.1 and 30 °C with 0.25 U/mL of purified enzymes.

The impact of the mutations on the topology and dynamics of the active site for the mutants studied by MD simulations is clear (Fig. 6), and this behavior could thus affect the enthalpy component of the free binding energy. The perturbation of the enzyme structure by the introduction of mutations could also affect the pKa of the catalytic residues^37^. The entropic contribution to the binding energy could explain the loss of activity of the F2136G mutant. The free energy landscapes taken from the two eigenvectors from the MD simulations of the free ligand enzymes (Fig. 5) and in complex with sucrose (Supplementary Fig. S7) revealed a tightened free energy landscape in the case of the mutant F2136G:sucrose complex indicating an important entropy loss in comparison to the free ligand FEL. This phenomenon was not as significant in the case of W2135S-F2136L and W2135I-F2136C mutants suggesting that kinetics parameters for these mutants could be affected rather by the enthalpy contribution of the binding energy.

## Conclusion

Overall, our study shows how amino acid substitutions introduced in the acceptor subsites + 1, + 2 and + 3 of ΔN_123_-GBD-CD2 branching sucrase could drastically shift the preferred binding mode of a non-natural tetrasaccharide acceptor, a lightly protected precursor in the synthesis of *S. flexneri* haptens, and thereby control its regioselective glucosylation. From the screening of a focused library of 22 mutants earlier reported^6^, we identified 6 mutants exhibiting singular product profiles with respect to the parental enzyme which was earlier reported to produce in particular a pentasaccharide **P1** and an uncharacterized monoglucosylated product **P2**. By introducing a limited number of mutations—up to three—it was possible to drive the transglucosylation reaction toward one extremity or the other of the tetrasaccharide **ABC′D′** (**A** vs **D′**), producing four distinct pentasaccharides (**P1, P2**, **P2′**, and **P3**) in different amounts. Among these four products, two are characteristic of *S. flexneri* serotype-specific pentasaccharide repeating units (*S. flexneri* serotype 4a/4b for **P1** and the prevalent serotype 3a for **P2**). Interestingly, two of these pentasaccharides (**P2′** and **P3**) and an additional di-glucosylated tetrasaccharide (**H1**) were also not produced by parental ΔN_123_-GBD-CD2.

Molecular dynamics simulations were further carried out and analyzed in details using a graph approach that enabled to map distance matrices of active site residues along the simulation. The resulting amino acid residue networks computed for parental enzyme and its mutants revealed interesting features and specific patterns of residue interactions that helped to understand product profile specificity. Taken together, this information enabled to shed some light on the molecular and dynamical determinants responsible for the regioselectivity of the ΔN_123_-GBD-CD2-mediated tetrasaccharide transglucosylation and activity toward sucrose.

Given the diversity of products obtained upon introduction of just a few mutations (up to 3) in the very exposed active site of ΔN_123_-GBD-CD2, having more than 50 residues within 10 Å radius of the sucrose in its productive conformation, one can hope that purposely re-designing branching sucrase active site using computer-aided engineering methods could enable further glucodiversification and improve enzyme catalytic efficiency. Such strategies will undoubtedly provide opportunities in the near future to develop biocatalysts needed for the development of chemo-enzymatic routes toward *S. flexneri* serotype-specific haptens that could enter in the composition of broad coverage vaccines.

## Material and methods

### Tetrasaccharide acceptor

The **ABC′D′** acceptor was synthesized as described^8^.

### Bacterial strains, plasmids and chemicals

The library of mutants was generated in *E. coli* as previously reported^6^. Briefly, *E. coli* BL21 Star DE3 were transformed by pET53 plasmids containing the genes of the ΔN_123_-GBD-CD2 parental enzyme or its mutants. Twenty milliliters of LB-Miller medium, supplemented with ampicillin (100 μg mL^−1^), were inoculated with 100 μL of transformation mix and incubated overnight at 37 °C under agitation (200 rpm). All chemicals used in the study were of analytical grade.

### Production of the library of ΔN123-GBD-CD2 in flasks

Mutants were produced in 50 mL baffled Erlenmeyer flasks containing modified ZYM-5052 medium^38^ with 0.75% lactose, 0.05% glucose and 1.5% glycerol. Culture media supplemented with ampicillin (100 μg mL^−1^) were inoculated with a starter culture at an OD_600nm_ of 0.05. Cultures were incubated for 32 h at 23 °C, under agitation (150 rpm). The cells were then harvested by centrifugation and re-suspended in 50 mM sodium acetate buffer pH 5.75 at a final OD_600nm_ of 30. Cells were lyzed by sonication, and enzymes were recovered in the soluble fraction after centrifugation of the crude cell extract (15,500*g*, 30 min, 8 °C).

### Sucrose activity assay of the library of ΔN123-GBD-CD2 branching sucrases

After enzyme recovery in the soluble fraction, the activity on sucrose was assayed in 1 mL format using the DNS method^8^. One unit of activity is described as the amount of enzyme necessary to release one micromole of fructose in one minute at 30 °C and in sodium acetate buffer at 50 mM and considered pH (5.75, pH 5.1 or pH 4.7 as stated).

### Acceptor glucosylation reaction of the library of ΔN123-GBD-CD2 branching sucrases

The enzymatic glucosylation assays were performed in miniaturized format at 10 µL or 50 µL scale in glass inserts. The final concentrations of the acceptor **ABC′D′** and sucrose donor were 50 mM and 1 M, respectively. At 10 µL scale, 0.2–0.7 µL of sonicated crude enzyme extract was added that correspond to an activity between 0.003 and 0.037 U mL^−1^, depending on the enzyme and the reaction was performed in sodium acetate buffer 50 mM at a fixed pH of 4.7. For 50 µL reactions, 1 U mL^−1^ enzyme was added and the pH was fixed at 5.1 in sodium acetate buffer 50 mM. Reactions were stopped by diluting them 5 times in a solution of H_2_O/acetonitrile (ACN) (70:30) containing 0.08% of TFA. TFA was used to acidify the reaction mixture for product stabilization^8^. The reaction mixture was used for HPLC–UV-HRMS analysis.

### Analytical methods used for carbohydrate separation and detection

10 µL reactions and samples collected after product purification were analyzed by HPLC (Dionex UltiMate 3000, Thermo Scientific, San Jose, CA, USA) using a C18-RP Fusion analytical column (4 µm, 80 Å, 250 × 4.6 mm) coupled to a detection by UV at 220 nm and a low resolution mass spectrometry (MSQ Plus single quadrupole mass spectrometer, Thermo Scientific, San Jose, CA, USA). Mass of the different compounds was determined by mass spectrometry with a 0.5 s full scan (*m/z* 200–1950) both in positive and negative modes. Needle was set on 3.5 kV, cone on 60 V, and the probe temperature was maintained at 450 °C. The following elution method was applied: 20 min of separation with an isocratic step of H_2_O/ACN 70:30 from 0 to 4 min and a linear gradient H_2_O/ACN 70:30 to 40:60 from 4 to 11 min, followed by washing and re-equilibrations steps. The flow was kept at 1 mL min^−1^ and the column at 40 °C.

Reactions performed at 50 µL scale with ΔN_123_-GBD-CD2 enzyme and mutants W2135I-F2136C, W2135S-F2136L and F2163G were analyzed using HPLC–UV coupled with high resolution mass spectrometry with a separation method previously described^8^.

LC HRMS analyses of the products were carried out on an LC–MS platform composed of a Thermo Scientific Dionex Ultimate 3000 system, coupled to a Thermo Scientific LTQ Orbitrap Velos hybrid ion trap-orbitrap mass spectrometer (Thermo Fisher Scientific, San Jose, CA, USA). Samples were diluted 5 times in a mixture H_2_O/ACN 70:30 with 0.08% TFA prior to injection. The liquid chromatography was performed on C18-RP Fusion column, 250 mm × 4.6 mm; 4 µm, 80 Å. The column was kept at 40 °C and the flow rate was set to 1 (mL.min^-1^. The solvent system consisted of (A): H_2_O and (B): ACN. The gradient program was as following: 46 min of separation with an isocratic step of (A)/(B) = 80:20 (v/v) from 0 to 15 min and a linear gradient (A)/(B) = 80:20 (v/v) to 20:80 from 15 to 30 min. This ratio was maintained for 5 min and was a re-equilibration step at (A)/(B) = 80:20 (v/v) during 11 min. The injected sample amount was 10 µL and autosampler temperature was kept at 4 °C. Mass detection was carried out in a positive heated electrospray ionization (HESI) mode. Conditions for the ESI positive ion mode were as follows: source voltage 4.0 kV; capillary temperature 350 °C; source heater temperature 350 °C; sheath gas (nitrogen) flow rate, 75 arb; auxiliary gas flow rate, 20 arb; S-lens RF level, 70%. For the full scan MS analysis, the spectra were recorded in the range of *m/z* 500–1900 and the FT resolution at 60,000 (FWHM).

### Production and purification of enzymes

ΔN_123_-GBD-CD2, and mutants W2135S-F2136L and F2163G were produced in 1 L scale using the same method described as above but during 24 h instead of 32 h in order to avoid accumulation of aggregated or degraded forms. Purification was then performed by affinity chromatography onto nickel resins as previously described^8^. Protein fractions were collected and controlled by sodium dodecyl sulfate–polyacrylamide gel electrophoresis (SDS-PAGE) as previously described^8^.

### Synthesis of pentasaccharide products **P2** and **P2′**

Synthesis of the mixture of **P2**/**P2′** mono-glucosylated products was achieved in a 2 mL scale reaction at pH 5.1 using 1 U mL^−1^ of purified F2163G mutant with 50 mM **ABC′D′** acceptor (88 mg) and 1 M sucrose donor (685.96 mg), during 16 h of reaction. After stopping of the reaction (dilution 5 times in H_2_O/ACN + 0.08% TFA) the sample was analyzed in HPLC–UV, then purified using an automated fraction collector. After re-analysis of the fractions by HPLC–UV, the ones containing pure product **P2/P2′** were pooled, acetonitrile was evaporated using a rotavap, and the samples were frozen at − 80 °C and lyophilized prior to re-suspension in D_2_O and NMR analyses.

In a second synthesis performed in a 1.5 mL scale (66 mg acceptor and 514.47 mg sucrose), two rounds of purification were performed in order to isolate **P2′** from the mixture **P2/P2′**. The reaction was stopped by diluting the sample 2 times in H_2_O/ACN (40:60) + TFA 0.16%. Modifications were applied to the elution method of the second round of purification, first an isocratic step was applied at 20% of acetonitrile during 30 min then a 10 min gradient was applied to reach 60% acetonitrile, and the column was re-equilibrated for 11 min at 20% acetonitrile. After automated collection and re-analysis by HPLC–UV, the fractions containing pure product were evaporated to dryness using a SpeedVac, and re-suspended in D_2_O prior to NMR analyses.

### NMR experiments

The samples were dissolved in DCl-containing D_2_O at pH 5.1. For NMR studies, the samples were lyophilized three times and dissolved in 180 µL of 99.9% DCl-containing D_2_O.

All NMR spectra were recorded on a Bruker Avance spectrometer operating at a proton frequency of 950 MHz (TGIR- RMN-THC Fr3050 CNRS, Gif-sur-Yvette) and at a carbon frequency of 238 MHz with a 5-mm gradient indirect cryoprobe. All spectra were processed and analyzed with Topspin software (Bruker) and assigned using SPARKY (T. D. Goddard and D. G. Kneller, SPARKY 3, University of California, San Francisco).

^1^H and ^13^C 1D NMR spectra were accumulated at 25 °C, 65,536 data points were acquired with 32 and 2048 scans respectively for proton and carbon experiments. ^1^H–^13^C HSQC (Heteronuclear Single Quantum Coherence spectroscopy), HMBC (Heteronuclear_single_quantum_coherence_spectroscopy) with *J*_CH_ log range of 3.5 Hz, and Double Quantum Filtered COrrelation SpectroscopY (QDF COSY)^15^ experiments were performed at 25 °C. Homo and heteronuclear spectra were recorded under the following experimental conditions: 512 increments of 2048 complex points are acquired with an accumulation of 16 scans. Spectral widths were 16,025 Hz for protons dimension and 44,267 Hz for carbon dimension.

### Kinetic parameter determination

Kinetic parameters on sucrose were determined at pH 5.1 (buffered with sodium acetate at 50 mM) and 30 °C using 0.25 U mL^−1^ of purified enzymes. Sucrose was used in increasing concentrations ranging from 0 to 300 mM. Reactions were stopped by heating the samples at 95 °C for 2 min and analyzed by HPLC-CAD on HPX-87C columns. The k_cat_, V_max_ and K_m_ values were determined using Michaelis–Menten equation.

### Computational procedures

MD simulations were performed with the AMBER ff14SB^16^ force-field for enzymes and GLYCAM_06j-1^17^ for sugar ligands. Carbohydrate oligomers were constructed using tleap program from AMBER software package^27^. The protecting groups; a chloroacetyl ester at position 2 of the α-l-rhamnopyranosyl residue **C′** as well as the allyl aglycon and *N*-trichloroacetyl moiety at position 1 and 2 of the β-d-glucosaminide unit (**D′**), respectively, were constructed by Avogadro software^28^, the *N*-trichloroacetyl group of **D′** was obtained through substitution of the acetyl hydrogens by chlorine atoms. Quantum mechanical calculations were performed using the Gaussian software package. Geometries were optimized using the Gaussian default optimization criteria. The HF/6-31G* level of theory was employed. Resulting partial charges were fitted using restrained electrostatic potential (RESP)^29^ with 0.01 charge restraint weight^30^. The valence bond, angle, and torsional parameters of protecting groups were derived from gaff force field. Nevertheless, original GLYCAM geometrical parameters of sugar rings were conserved.

The 3D model for the parental enzyme was based on ΔN_123_-GBD-CD2 pdb entry: 3TTQ^10^. Mutants F2163G, W2135S-F2136L and W2135I-F2136C were constructed using Rosetta3 software^31^. The X-ray conformations were conserved for all amino acids except for the residues targeted by mutation for which the side chain rotations were permitted. The H++ webserver was used to determine the protonation state of ionisable residues at pH 5.75 at which the activity of ΔN_123_-GBD-CD2 and its mutants was experimentally tested. The enzyme:sucrose complexes were built by superposing ΔN_123_-GBD-CD2 and its variants to the inactive mutant of GTF180, pdb entry: 3HZ3 containing sucrose in the active site^26^. Furthermore, this sucrose binding structural knowledge was used for docking pentasaccharides; **ABC[E(1 → 6)]D′** and **[E(1 → 3)]ABC′D′** (**P1** and **P2**, respectively) in the active site. The strategy consisted on taking the coordinates of the glucosyl moiety from sucrose for the glucosyl of the pentasaccharides. Then, the simulated annealing (from 0 to 350 k in 10 ps and vice versa) of the systems in vacuum with harmonic positional restraints of 50.0 kcal/mol/Å^2^ on the enzyme and glycosyl unit, and the sugar pucker rings of **ABC′D′** was carried out for each system.

MD simulation were performed using the NAMD program^18^ for the Apo forms. The complex systems MD simulations were done by pmemd.CUDA^19^ supporting the mixed scaling of 1–4 non-bond electrostatic and van der Waals interactions^20^ of amino acids and sugars.

MD simulations were performed at constant temperature (303 K) and pressure (1 bar) using the Berendsen algorithm^21^. The integration time-step was 2 fs and covalent bonds involving hydrogen were constrained using SHAKE^22^. The non-bonded pair-list was updated heuristically. Long-range electrostatic interactions were treated using the particle mesh Ewald (PME) approach^23^. Non-bonded interactions were treated with a 9 Å direct space cut-off. All enzyme systems were neutralized with Na^+^ ions^24^ (minimal salt condition), in explicit TIP3P water molecules^25^; the primary boxes were rectangular with solvent extending 10 Å around the enzymes. The water molecules and counterions were energy-minimized and equilibrated at 100 K around the constrained solute for 100 ps in the NVT ensemble; the entire system was then heated incrementally over 100 ps from 100 to 300 K in 5 K steps with harmonic positional restraints of 25.0 kcal/mol/Å^2^ on the solute atoms. The MD simulations were continued in NPT, without notable change in volume. The positional restraints were gradually removed over 250 ps and followed by the production phase. MD snapshots were saved every 1 ps. The lengths of MD simulations were 1 μs for the free ligand forms, 100 ns for enzyme:sucrose complexes and 2 ns for enzyme:pentasaccharides complexes.

Principal Component Analyses (PCA), Root Mean Square Deviation (RMSD), B-factors, Free-energy landscape (FEL) analysis were carried out as described in our previous study of ΔN_123_-GBD-CD2 branching sucrose^11^. The active site residues average distance matrices from MD simulation were built using cpptraj^35^. The latter distance matrices were employed for the graph analyses based on the Fruchterman-Reingold algorithm^32^ and the spin-glass algorithm^33^ implemented in R software^39^ by Borsboom group.

Graphs in Fig. 6 were drawn using the R software^39^. Molecular graphics were prepared using PYMOL 1.7 (PyMOL Molecular Graphics System, Schrödinger, LLC).

## Supplementary Information

Supplementary Information.

## Data Availability

All the data generated and analyzed is available in this published article or in its Supplementary information. This available data may be requested from the corresponding authors.
