# Supplementary material for: Redirecting substrate regioselectivity using engineered ΔN123-GBD-CD2 branching sucrases for the production of pentasaccharide repeating units of S. flexneri 3a, 4a and 4b haptens
Source: Sci Rep. 2021 Jan 28;11:2474. doi: 10.1038/s41598-021-81719-1 (PMC7844235; doi:10.1038/s41598-021-81719-1)
Supplement: Supplementary file 1 — Supplementary Information. [file 41598_2021_81719_MOESM1_ESM.docx]

**Supplementary Information**

**Redirecting substrate regioselectivity using engineered ΔN_123_-GBD-CD2 branching sucrases for the production of pentasaccharide repeating units of *S. flexneri* 3a, 4a and 4b haptens**

Mounir Benkoulouche^1#^, Akli Ben Imeddourene^1#^, Louis-Antoine Barel^2,3^, Guillaume Le Heiget^2,3,4^, Sandra Pizzut-Serin^1^, Hanna Kulyk^1,5^, Floriant Bellvert^1,5^, Sophie Bozonnet^1^, Laurence A. Mulard^2^, Magali Remaud-Siméon^1^, Claire Moulis^1^* & Isabelle André^1^*

*^1^ Toulouse Biotechnology Institute, TBI, Université de Toulouse, CNRS, INRAE, INSA, Toulouse, France. 135, avenue de Rangueil, F-31077 Toulouse Cedex 04, France
^2^ Unité de Chimie des Biomolécules, Institut Pasteur, UMR3523 CNRS, 28 rue du Dr Roux, 75724 Paris Cedex 15, France*

*^3^ Université Paris Descartes, Sorbonne Paris Cité, Paris, France
^4^ Université Paris 13, Bobigny, France*

*^5^  MetaboHUB-MetaToul, national infrastructure for metabolomics and fluxomics, Toulouse, France*

# Both authors contributed equally

* Corresponding authors: [claire.moulis@insa-toulouse.fr](mailto:claire.moulis@insa-toulouse.fr); [isabelle.andre@insa-toulouse.fr](mailto:isabelle.andre@insa-toulouse.fr)

**Supporting Figures**

**
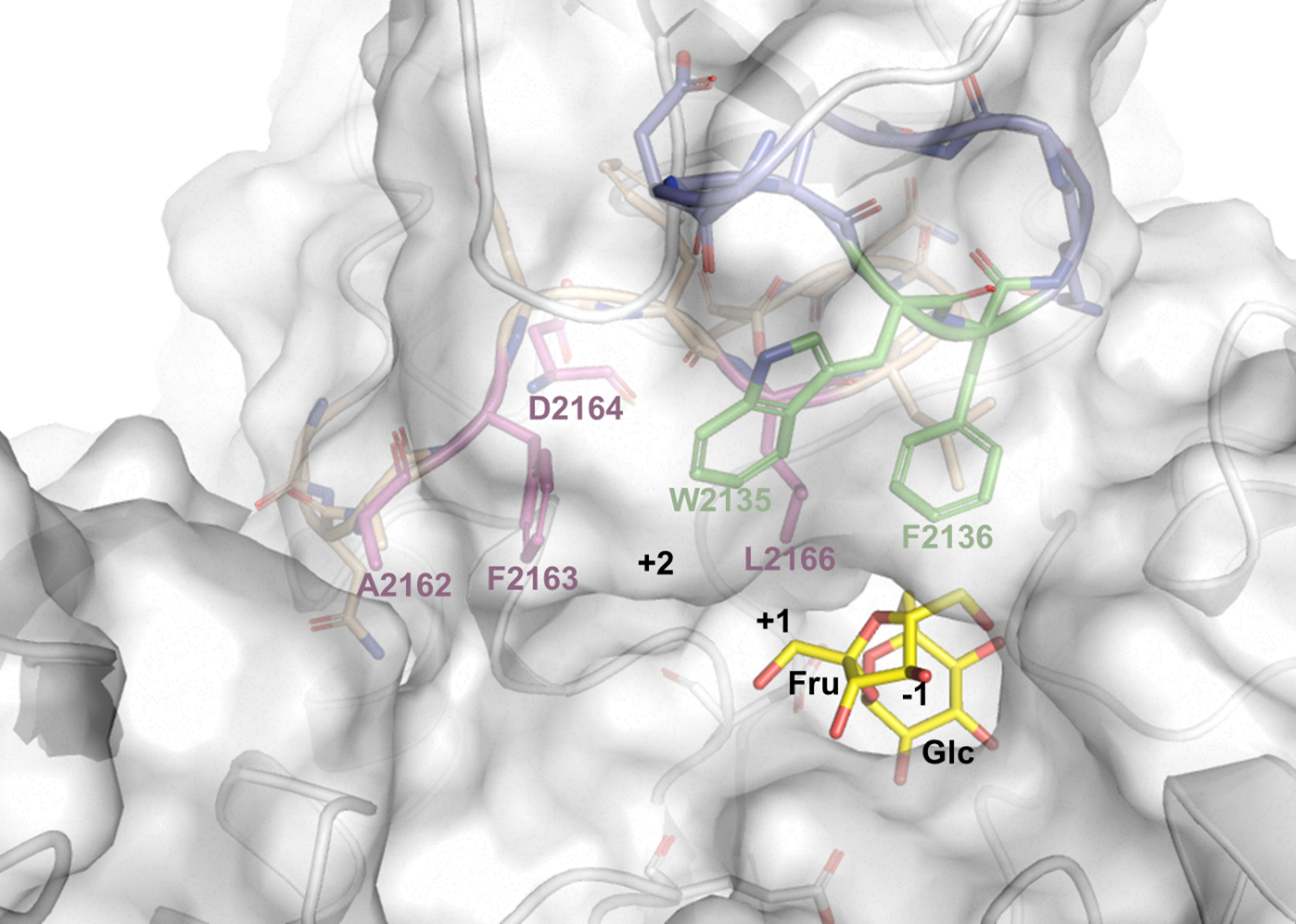
**

**Figure S1.** View of ΔN_123_-GBD-CD2 active site. Donor (-1) and acceptor (+1, +2) subsites are represented. The targeted residues in the collection of 22 mutants are displayed as sticks (W2136, F2136, A2162, F2163, D2164, L2166). Sucrose is shown for reference purpose and was taken from^1^.


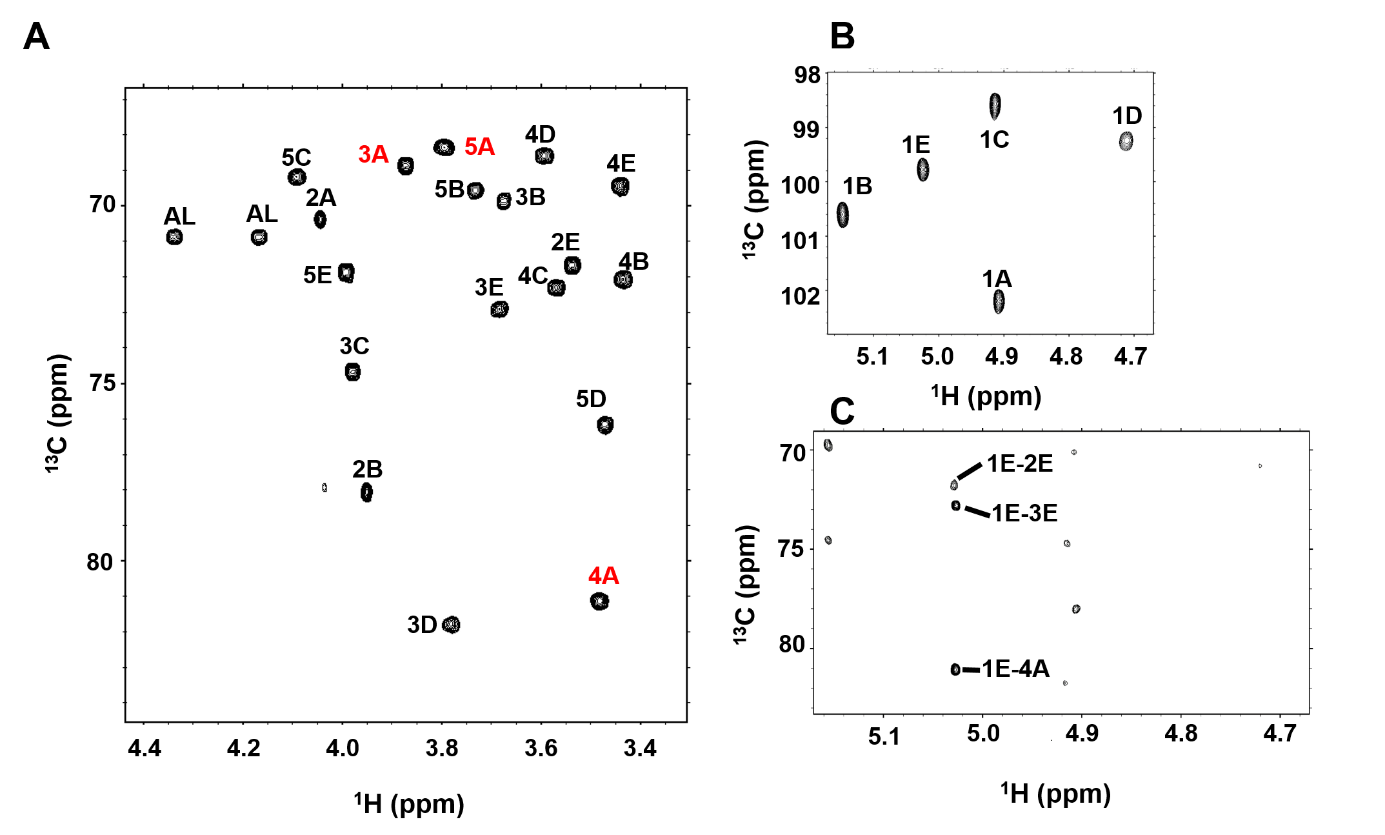


**Figure S2.** ^1^H-^13^C HSQC spectra of the C2, C3, C4 and C5 resonances (A) and the anomeric region (B) of **P2’** pentasaccharide. The ^1^H-^13^C HMBC illustrates the correlation between the **E** and **A** units (C). The shifted resonances, compared to the tetrasaccharide, were labeled in red color. All spectra were acquired at 950 MHz.


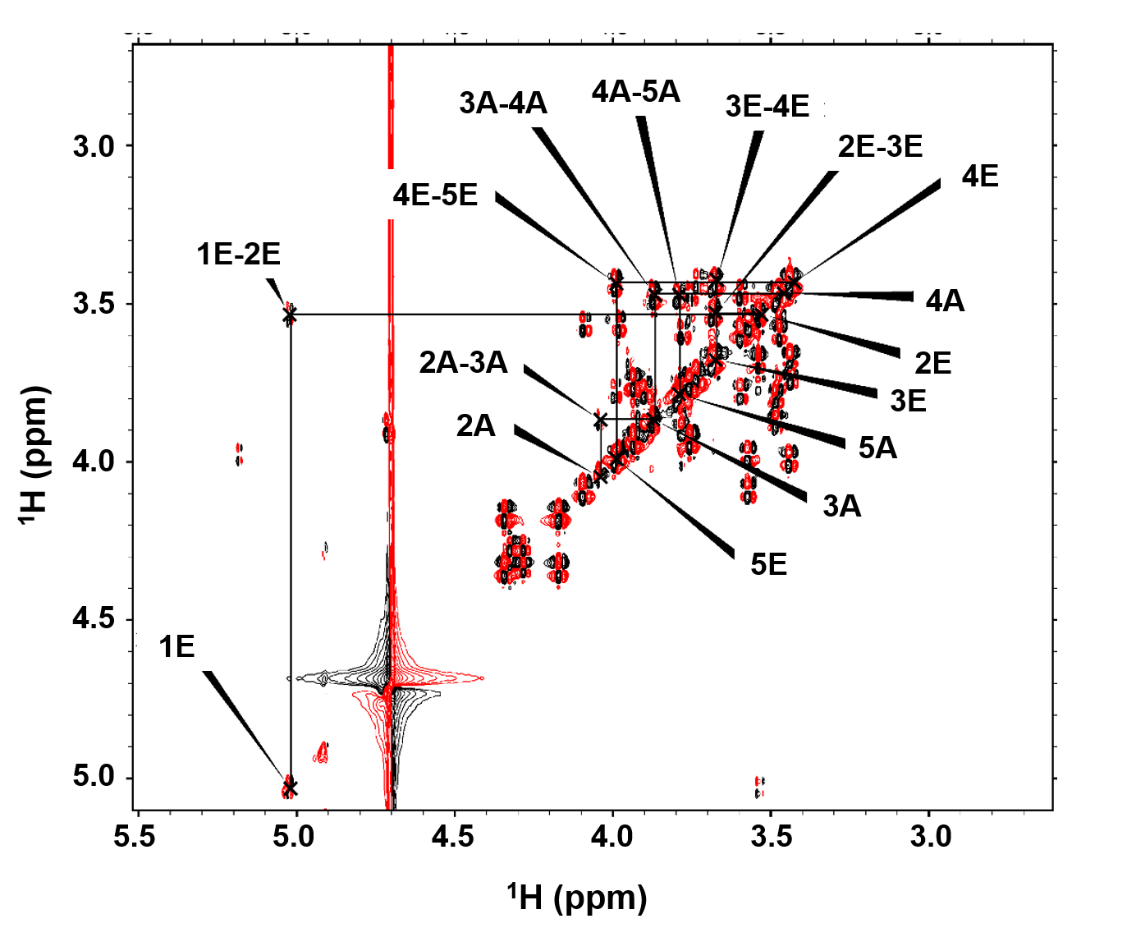


**Figure S3.** ^1^H-^1^H DQF COSY spectrum at 950 MHz of the **P2’** pentasaccharide. Positive and negative peaks are showed in black and red respectively. The **E** and **A** units connectivities are plotted and the corresponding peaks are labeled.

**
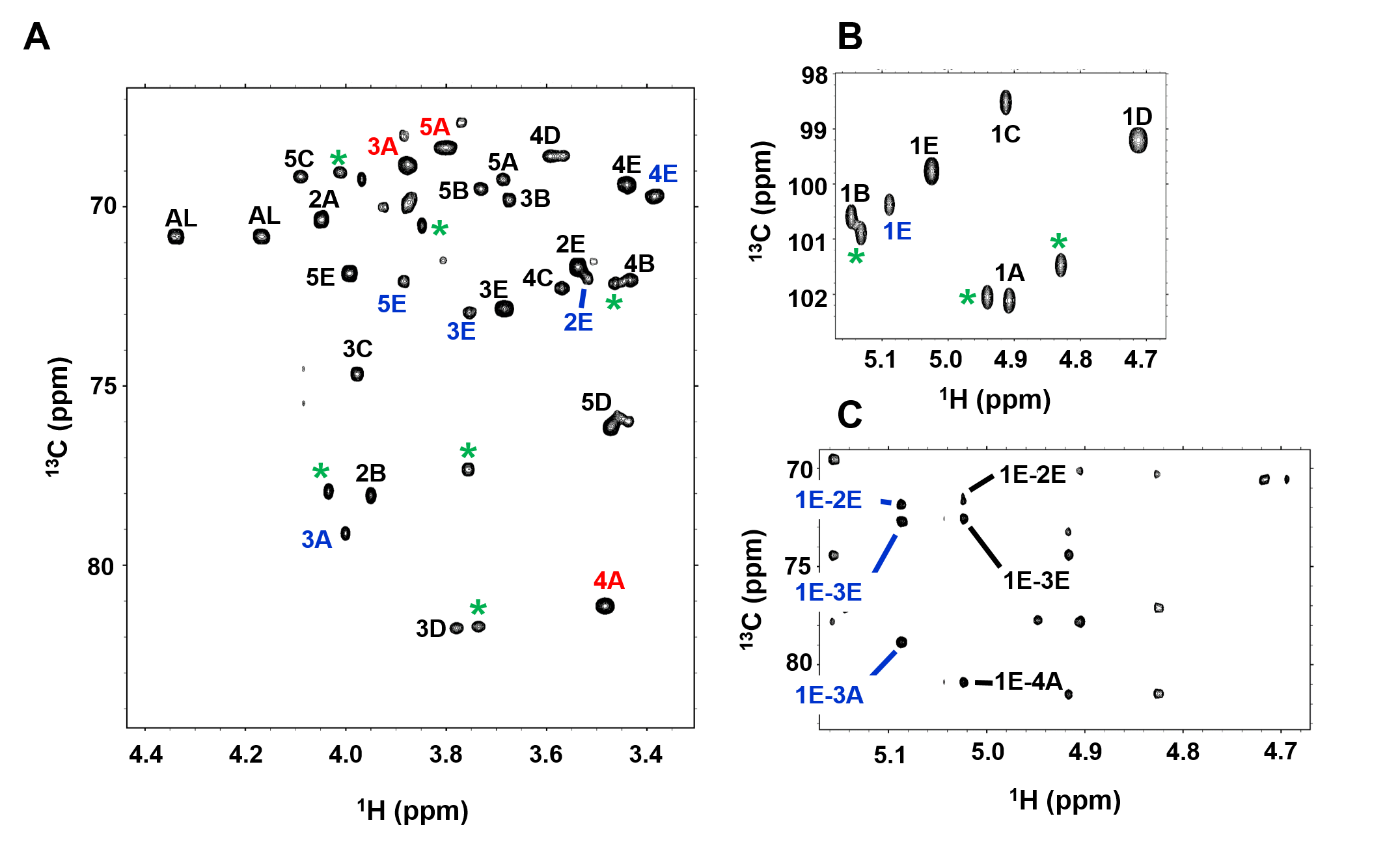
**

**Figure S4.** ^1^H-^13^C HSQC spectra of the C2, C3, C4 and C5 resonances (A) and the anomeric region (B) of the mixture. The ^1^H-^13^C HMBC illustrates the correlation between the **E** and **A** units of **P2** and **P2’** (C). The shifted resonances of the **P2’**, compared to the tetrasaccharide, were labeled in red color and the **A** and **E** units belonging to the **P2** are highlighted by blue color. The peaks of the dechloroacetyled form **ABCD’** are labelled by green stars. All spectra were acquired at 950 MHz.


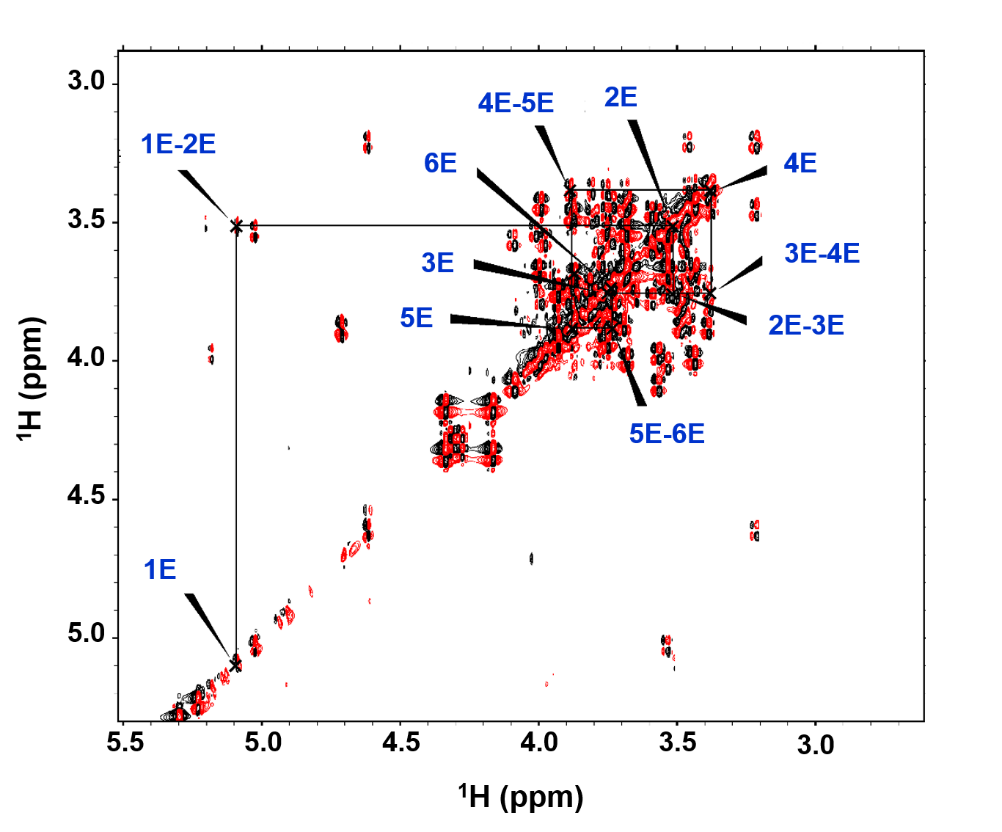


**Figure S5.** ^1^H-^1^H DQF COSY spectrum at 950 MHz of the mixture. Positive and negative peaks are showed in black and red respectively. The E units belonging to the **P2** connectivities are plotted and the corresponding peaks are labeled in blue color.


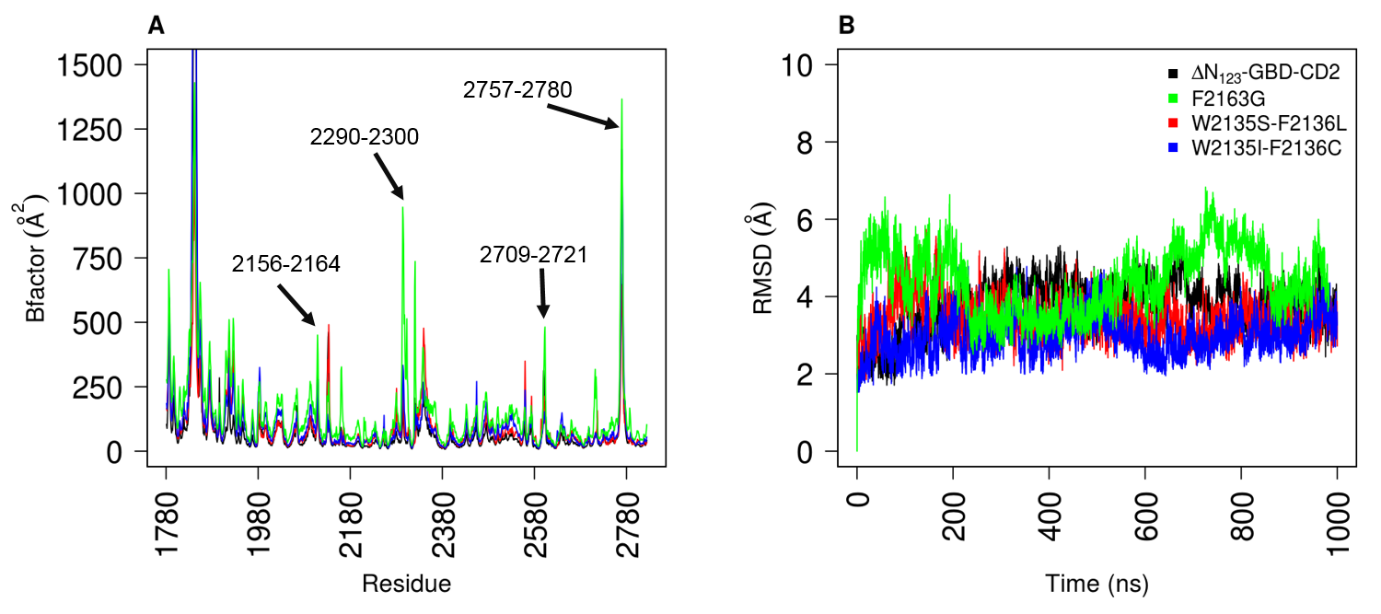


**Figure S6.** Analysis of MD simulations. **(A)**: B-factors were calculated as function of enzyme amino acid residues along 1µs of MD simulation of the parental ΔN_123_-GBD-CD2 (black), the mono mutant F2163G (green), and double mutants W2135I-F2136C (blue) and W2135S-F2136L (red). The regions pointed by the arrows are those discussed in the text. **(B)** Carbon α atoms Root Mean Square Deviation of ΔN_123_-GBD-CD2 (black line), W2135I-F2136C (red line), W2135S-F2136L (blue line) and F2163G (green line), with respect to parental ΔN_123_-GBD-CD2 X-ray structure (PDB code: 3TTQ) were calculated as a function of simulation time


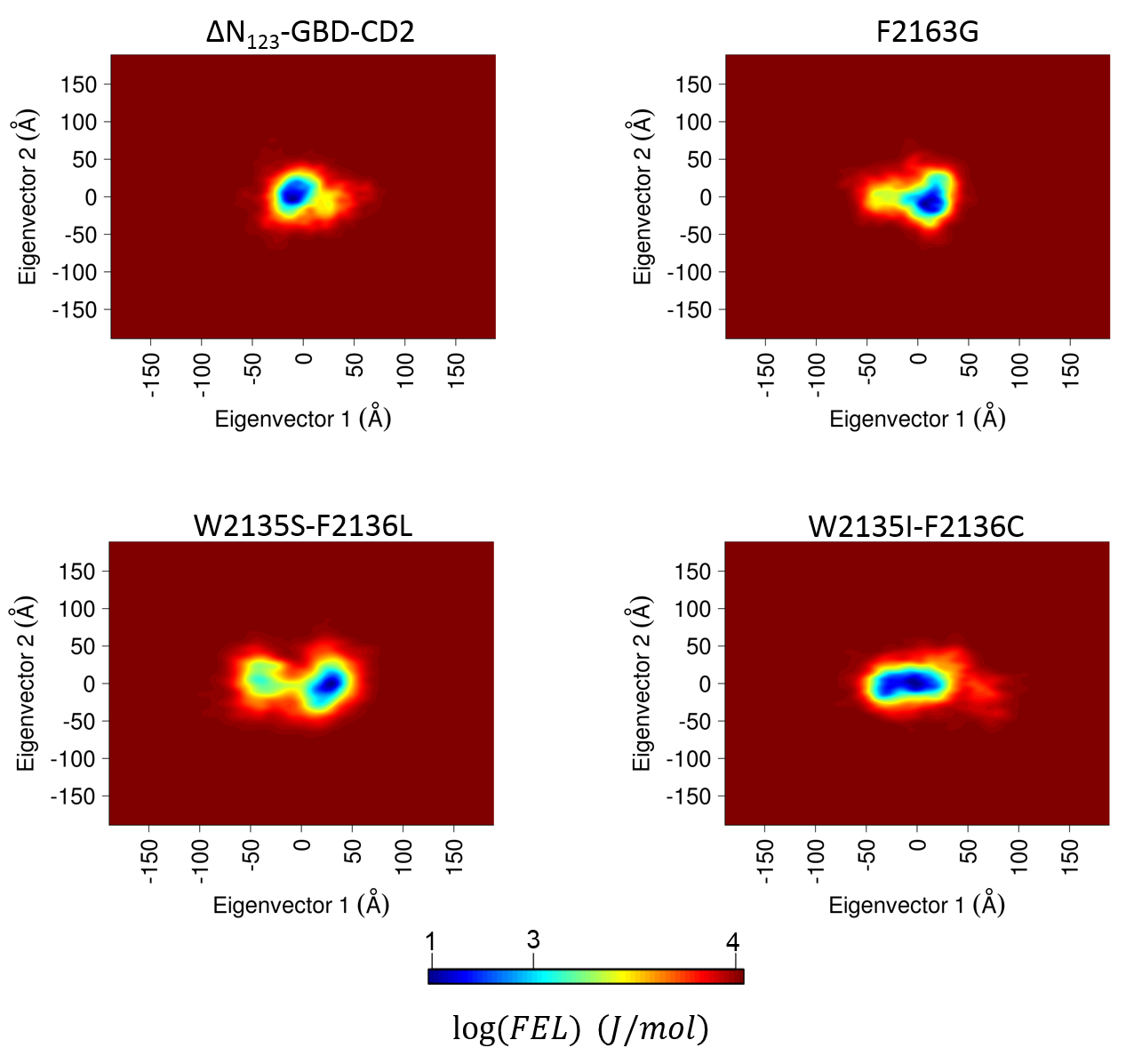


**Figure S7.** Free energy landscape (FEL) of enzyme:sucrose complexes of parental ΔN_123_-GBD-CD2, F2163G, W2135S-F2136L and W2135I-F2136C were determined using as reaction coordinates the projection of the first and second principal components from 100 ns MD simulation of the enzyme:sucrose complexes for each system. The bottom legend shows the color scale of the logarithm of FEL in J.mol^-1^.

**
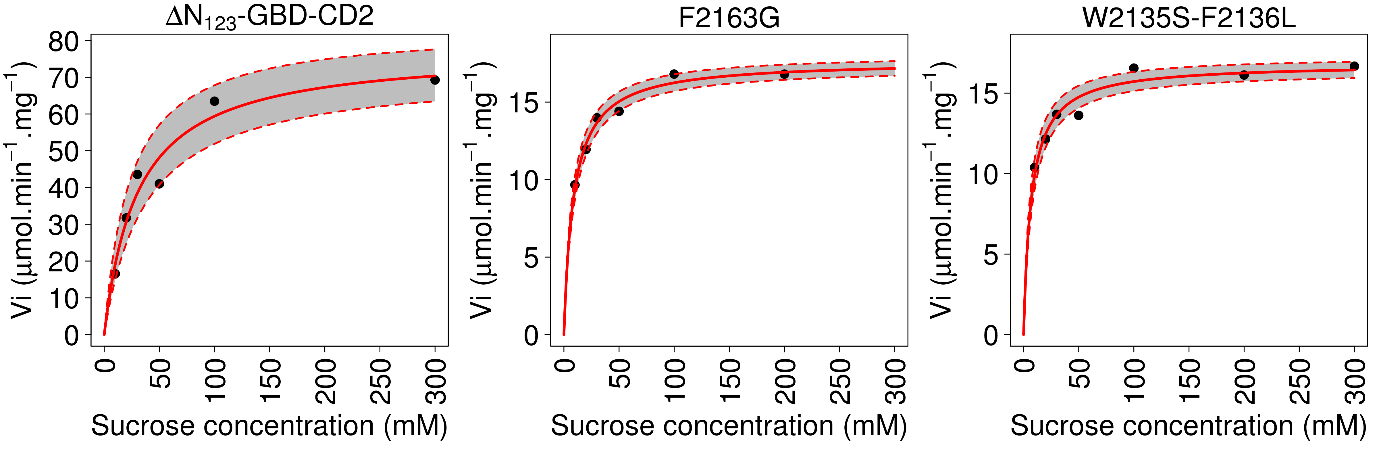
**

**Figure S8.** Initial velocities of sucrose hydrolysis activity of parental ΔN_123_-GBD-CD2, and mutants F2163G and W2135S-F2136L. Determination of kinetic parameters was performed using increasing concentrations of sucrose (ranging from 10 to 300 mM), at pH 5.1 and 30 °C with 0.25 U.mL^-1^ of purified enzymes. The curves obtained by the least square fit are represented by the red continuous line and residual standard deviations are depicted by dashed red lines and the area between them is highlighted by gray color.

**Supporting Tables**

**Table S1.** The parameters extracted from the fitted Michaelis-Menten equation equation of N number experimental data having R² correlation coefficient with fit P-value. The K_M_ and V_max_ are given with their standard deviation. The fisher values versus the hypothesis of linear fits calculated were significantly higher than the F-table values for ν_1_ = 1 and ν_2_ = 4 or 3 from F-table provided between brackets for α = 0.05

| Enzyme | N | R² | K_M_ | V_max_ | P-value | F-stat |
| --- | --- | --- | --- | --- | --- | --- |
| ΔN_123_-GBD-CD2 | 6 | 0.95 | 30.5±7.2 | 77.5±6.1 | 0.001 | 71.0 (7.7) |
| W2135S-F2136L | 5 | 0.97 | 8.8±1.0 | 17.7±0.4 | 0.0002 | 151.9 (10.1) |
| F2163G | 6 | 0.93 | 7.0±1.2 | 16.8±0.5 | 0.0004 | 67.8 (7.7) |

**Supporting references**

1. Ben Imeddourene, A., Esque, J. & André, I. Combining multi-scale modelling methods to decipher molecular motions of a branching sucrase from glycoside-hydrolase family 70. *PLoS One* **13**, e0201323 (2018).
